# STAT3-induced long noncoding RNAs in multiple myeloma cells display different properties in cancer

**DOI:** 10.1038/s41598-017-08348-5

**Published:** 2017-08-11

**Authors:** Stefanie Binder, Nadine Hösler, Diana Riedel, Ivonne Zipfel, Tilo Buschmann, Christoph Kämpf, Kristin Reiche, Renate Burger, Martin Gramatzki, Jörg Hackermüller, Peter F. Stadler, Friedemann Horn

**Affiliations:** 1 Institute of Clinical Immunology, Faculty of Medicine, University of Leipzig, Leipzig, Germany; 2 Fraunhofer Institute for Cell Therapy and Immunology, Department of Diagnostics, Leipzig, Germany; 3 The RIBOLUTION Consortium, Leipzig, Germany; 4 Young Investigators Group Bioinformatics and Transcriptomics, Department Proteomics, Helmholtz Centre for Environmental Research - UFZ, Leipzig, Germany; 5 Department of Computer Science, University of Leipzig, Leipzig, Germany; 6 Bioinformatics Group, Department of Computer Science, University of Leipzig, Leipzig, Germany; 7 LIFE – Leipzig Research Center for Civilization Diseases, University of Leipzig, Leipzig, Germany; 8 Division of Stem Cell Transplantation and Immunotherapy, Department of Internal Medicine 2, Christian-Albrechts-University, Kiel, Germany; 9 Interdisciplinary Center for Bioinformatics, University of Leipzig, Leipzig, Germany; 10 German Centre for Integrative Biodiversity Research - iDiv, Halle-Jena-Leipzig, Germany; 11 Max Planck Institute for Mathematics in the Sciences, Leipzig, Germany; 12 Department of Theoretical Chemistry, University of Vienna, Vienna, Austria; 13 Center for RNA in Technology and Health, University of Copenhagen, Copenhagen, Denmark; 14 Santa Fe Institute, Santa Fe, USA

## Abstract

Interleukin-6 (IL-6)-activated Signal Transducer and Activator of Transcription 3 (STAT3) facilitates survival in the multiple myeloma cell line INA-6 and therefore represents an oncogenic key player. However, the biological mechanisms are still not fully understood. In previous studies we identified microRNA-21 as a STAT3 target gene with strong anti-apoptotic potential, suggesting that noncoding RNAs have an impact on the pathogenesis of human multiple myeloma. Here, we describe five long noncoding RNAs (lncRNAs) induced by IL-6-activated STAT3, which we named STAiRs. While STAiRs 1, 2 and 6 remain unprocessed in the nucleus and show myeloma-specific expression, STAiRs 15 and 18 are spliced and broadly expressed. Especially STAiR2 and STAiR18 are promising candidates. STAiR2 originates from the first intron of a tumor suppressor gene. Our data support a mutually exclusive expression of either STAiR2 or the functional tumor suppressor in INA-6 cells and thus a contribution of STAiR2 to tumorigenesis. Furthermore, STAiR18 was shown to be overexpressed in every tested tumor entity, indicating its global role in tumor pathogenesis. Taken together, our study reveals a number of STAT3-induced lncRNAs suggesting that the interplay between the coding and noncoding worlds represents a fundamental principle of STAT3-driven cancer development in multiple myeloma and beyond.

## Introduction

Multiple myeloma is an aggressive and incurable cancer of plasma cells mostly within the bone marrow. Myeloma cells rely on the pleiotropic cytokine interleukin-6 (IL-6), which is hallmarked by a wide range of biological functions, including immune regulation, hematopoiesis, inflammation, and tumor development^1^. IL-6 operates as a pro-inflammatory and anti-apoptotic stimulus through an intracellular signaling cascade^2^. Binding of IL-6 to its plasma membrane receptor activates receptor-associated Janus kinases (JAKs), which in turn phosphorylate intracellular targets^3^ including Signal Transducer and Activator of Transcription 3 (STAT3). Phosphorylated STAT3 dimerizes and shuttles to the nucleus, where it activates transcription of target genes^4^. In multiple myeloma, the IL-6-triggered STAT3 signaling represents a pivotal oncogenic pathway that acts primarily through regulation of cell survival, rendering multiple myeloma an ideal model system to study STAT3 function^5, 6^. As reported earlier, the IL-6-dependent human myeloma cell line INA-6 responds with a remarkably rapid and complete apoptosis to cytokine withdrawal^6^ as well as STAT3 knockdown, proving that IL-6-activated STAT3 is an essential survival factor. To uncover how STAT3 triggers survival of tumor cells, we analyzed IL-6-induced transcription patterns in INA-6 cells and identified the oncogenic microRNA-21 as a STAT3 target and anti-apoptotic regulator^7, 8^. The results demonstrated the involvement of ncRNAs in myeloma cell survival. In a genome-wide transcription study conducted in INA-6 cells using tiling arrays we demonstrated that in addition to protein-coding mRNAs, IL-6 induces the transcription of a large number of long noncoding RNAs (lncRNAs)^9^. Here, we characterize some of these IL-6-induced lncRNAs in more detail, verifying them as STAT3 targets, and therefore termed them STAT3-induced ncRNAs (STAiRs). Our data further support the view that lncRNAs contribute to STAT3-dependent tumorigenesis in multiple myeloma as well as in other cancer types.

## Results

### Transcription of IL-6-induced long ncRNAs is mediated by STAT3

**Figure 1:**
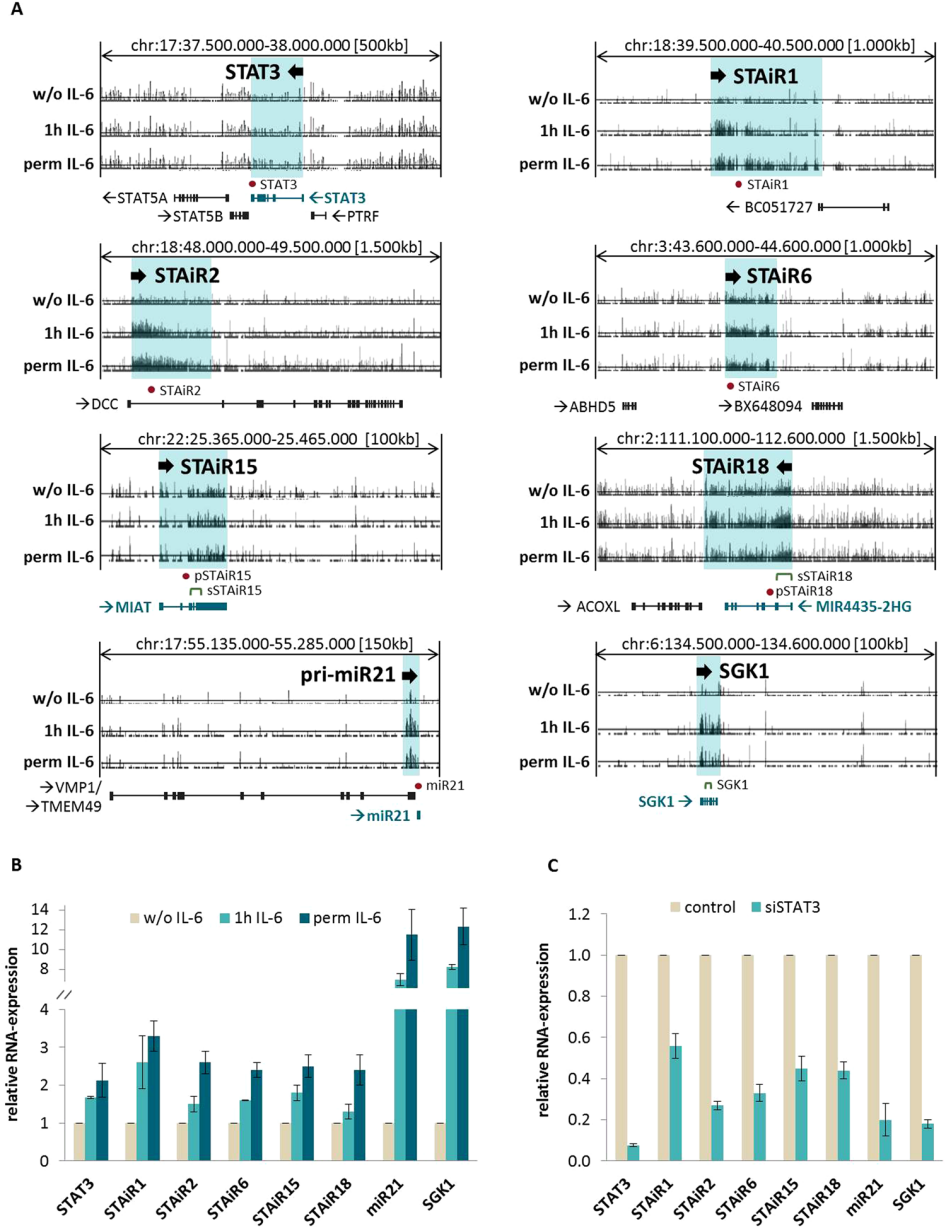
Identification and validation of STAT3-induced ncRNAs (STAiRs). (**A**) Expression profiles of STAiRs and control genes upon IL-6 treatment after tiling array analysis and mapping to the human genome hg18. INA-6 cells were either permanently grown in the presence of IL-6 (perm IL-6) or withdrawn from IL-6 for 12 h (w/o IL-6), and subsequently restimulated with IL-6 for 1 h (1 h IL-6). (**B**) Validation of STAiR-induction by IL-6. RNA isolated from INA-6 cells treated as described above was prepared, reverse transcribed and relative gene expression was determined by qPCR. (**C**) STAiR expression is STAT3-dependent. Permanently IL-6-treated INA-6 cells were transfected with either an siRNA targeting STAT3 or a control siRNA. The cells were harvested 24 h post-transfection and RNA was isolated. Relative gene expression was determined by RT-qPCR. In each case, the STAT3 knockdown led to significant reductions (p < 0.05) of target gene expression. For both, (**B**) and (**C**), primer pairs used for STAT3 and SGK1 control mRNAs are intron-spanning and detect spliced transcripts, whereas primers used for all STAiRs as well as the miR21 detect primary, unspliced transcripts. Expression values were first normalized to U6 RNA and then compared to the corresponding negative control. Data are expressed as mean ± SD (n ≥ 3).

In INA-6 multiple myeloma cells, we identified IL-6-induced long noncoding transcripts, as published previously^9^. RNA expression in IL-6-starved cells was used as a control, whereas starved cells with a subsequent 1-hour IL-6 restimulation and permanently IL-6-treated cells served to identify expression patterns of immediate-early and stably induced STAT3 target genes, respectively. After RNA preparation, samples were hybridized to tiling microarrays, which carried probes covering the non-repetitive part of the human genome (hg18). The study revealed a number of as yet unknown long noncoding RNAs induced by IL-6, amongst which we chose five transcripts for further analyses (see Fig. 1A and Table 1) based on their differential and significant expression strength upon IL-6 compared to the withdrawn control. Given that the JAK/STAT3 pathway is dominant in INA-6 cells upon IL-6 stimulation^5^ these transcripts were termed STAT3-induced ncRNAs (STAiRs). In general, with more than 20 kb in length, STAiRs refer to as macroRNAs. Except for STAiR2, which is transcribed from the first intron of the protein coding gene Deleted in Colorectal Cancer (DCC), the other STAiRs are expressed within intergenic regions. To date, STAiRs 1, 2, and 6 represent novel, not yet annotated transcripts, whereas STAiRs 15 and 18 match the already annotated ncRNA genes *MIAT* (also known as *Gomafu*) and *MIR4435-2HG*, respectively. As indicated by their expression profiles shown in Fig. 1A, STAiRs likely are long unprocessed RNAs. Furthermore, expression profiles of the known STAT3 target genes microRNA-21 (*miR21*), Serum/Glucocorticoid regulated Kinase 1 (*SGK1*), and the STAT3 locus itself show an induced expression upon IL-6, indicating a successful experimental setup.

**Table 1:** Selected STAT3-induced ncRNAs (STAiRs). Chromosomal coordinates and lengths of the transcribed regions are given referring to human genome hg18 and hg19.

STAiRs	hg18 coordinates	hg19 coordinates	Length	Strand	Position
STAiR1	chr18:39841200-40261200	chr18:41587202-42007202	420 kb	Plus	Intergenic
STAiR2	chr18:48174300-48474300	chr18:49920302-50220302	300 kb	Plus	Intronic
STAiR6	chr3:44009300-44139300	chr3:44034296-44164296	130 kb	Plus	Intergenic
STAiR15	chr22:25382800-25402800	chr22:27052800-27072800	20 kb	Plus	Intergenic
STAiR18	chr2:111584000-111974000	chr2:111867529-112257529	390 kb	Minus	Intergenic

Following their identification, the IL-6-induced expression of STAiRs was analyzed by qPCR (Fig. 1B). Although the expression profiles of STAiRs after 1-hour and permanent IL-6 stimulation (Fig. 1A) are comparable the validation demonstrated a higher expression for permanently treated cells (Fig. 1B). This confirms that these transcripts are not immediate-early but rather stably IL-6-enhanced genes. In fact, also mRNA targets of IL-6 in multiple myeloma divide into immediate early and late responders^6^. In general, STAiR expression was induced by IL-6 approximately 2-fold, comparable to the induction of STAT3 mRNA. In contrast, the already known STAT3 target genes *miR21* and *SGK1* show a higher fold-change of induction. However, most long ncRNAs are expressed less abundantly compared to mRNAs^10^. Additionally, a STAT3 knockdown proved that the expression of STAiRs is indeed STAT3-dependent. Here, STAiRs 2 and 6 showed a reduction of expression comparable to those of already known STAT3 target genes, *miR21* and *SGK1*. The expression of the remaining STAiRs was diminished to different extents by approximately 50%, suggesting their expression to be influenced by additional transcription factors (Fig. 1C). Taken together, STAiRs represent novel macroRNAs whose expression is induced by the transcription factor STAT3 upon IL-6 treatment.

### Subcellular localization and chromatin-association studies reveal a potential role of STAiR18 as epigenetic regulator

**Figure 2:**
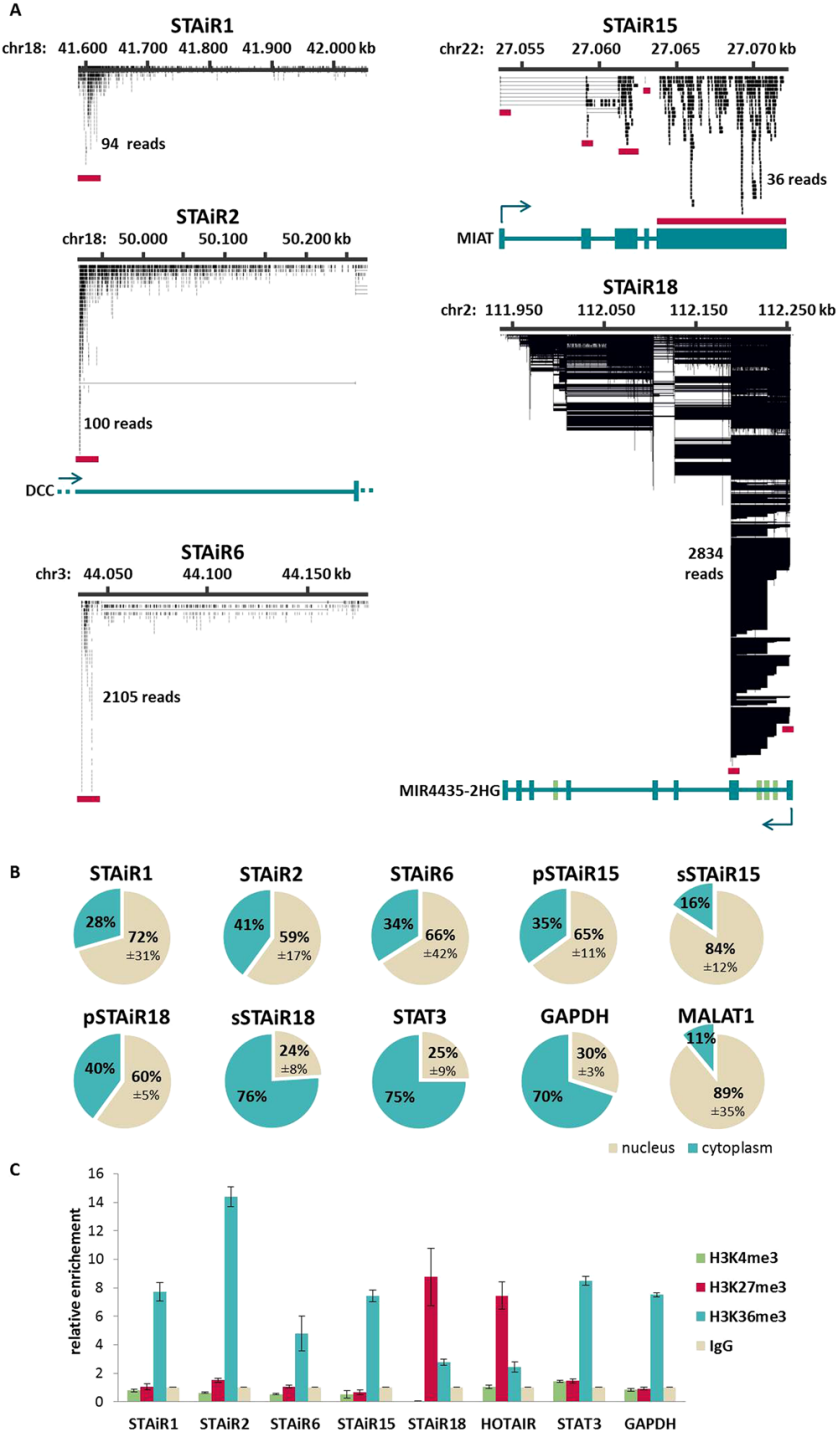
Processing, subcellular localization and chromatin association of STAiRs. (**A**) CAPTURE-RNA-sequencing was performed using 12 biotinylated oligonucleotides per STAiR target RNA (STAiRs 1, 2, 6, 15, 18) and 6 oligos for bacterial lacZ as a negative control. The pulldown was performed with RNA from permanently IL-6 stimulated (10 ng/ml) INA-6 cells. The RNA pulldown was implemented by streptavidin beads, following an RNA preparation, DNase digestion, library preparation (Scriptseq, Epicenter), and subsequent NGS. Identified reads were mapped to the human genome hg19. The resulting transcription patterns were visualized using Integrative Genome Viewer (IGV). The regions of oligo binding are marked with red lines below. For STAiRs 15 and 18 the annotated ncRNAs are shown in blue at the bottom. For STAiR18, 4 novel exons were identified shown in green. (**B**) Subcellular localization of STAiRs. Nuclear-cytoplasmic fractioning was performed with permanently IL-6-treated INA-6 cells. RNA was prepared, DNase-digested, reverse transcribed and relative gene expression determined by qPCR. For unspliced STAiRs 1, 2 and 6, the used primers detect primary transcripts, whereas for spliced STAiRs 15 and 18, both a pair detecting the primary (p) and spliced (s) transcript were applied. Primer pairs for STAT3 and GAPDH are intron-spanning. For detection of infrequently spliced MALAT1 a pair of exonic primers was used. Values were normalized to the corresponding cytoplasmic fraction. Means ± SD (in %) of STAiR expression per fraction are shown (n ≥ 3). (**C**) Chromatin association of STAiRs. RNA immunoprecipitation was performed with permanently IL-6-treated INA-6 cells using antibodies targeting H3K36me3, H3K4me3, and H3K27me3 as well as IgG as a negative control. RNA was prepared, DNase-digested, and reverse transcribed. RNA enrichment was analyzed by qPCR using intron-spanning primers (STAT3, GAPDH) and primers detecting the primary, unspliced ncRNA transcripts (STAiRs and HOTAIR). Samples were normalized to the IgG control. Means ± SD of STAiR enrichment per IP are shown (n ≥ 3).

To further investigate whether STAiR macroRNAs are processed to smaller transcripts, a CAPTURE-RNA-sequencing was performed. For this purpose, 12 biotinylated antisense oligonucleotides targeting each STAiR at positions indicated by red lines in Fig. 2A were used to pull down transcripts without prior fragmentation of nucleic acids isolated from permanently IL-6-treated INA-6 cells. Oligos complementary to STAiRs without annotation (STAiRs 1, 2, and 6) were designed to target the 5-prime regions, and oligos for STAiRs identified in genomic regions with an annotation (STAiR15/*MIAT* and STAiR18/*MIR4435-2HG*) were designed to target exons. The analysis of splice patterns was carried out after pull down by Next-Generation (RNA) Sequencing and identified reads were mapped to the human genome (hg19). For STAiRs 1, 2, and 6, the study revealed no split reads containing splice junctions. Thus, these data do not indicate any splicing event. Additionally, the position targeted by oligonucleotides was most strongly represented by the read coverage (see Fig. 2A). Regarding their enormous length, shear forces may be a reason for the decrease of read coverage in distal areas. As expected on the basis of annotation, STAiRs 15 and 18 showed transcript processing by splicing. Here, the enrichment of spliced transcripts was favored due to exonic oligo binding sites and the processed transcripts were enriched with a steady 5-to-3-prime distribution. Furthermore, pulldowns of STAiRs 15 and 18 exposed reads containing splice junctions and resemble the annotated transcripts of MIAT and MIR4435-2HG, respectively. For STAiR18 four novel exons marked in green could be identified in INA-6 cells, of which three are placed downstream to the first annotated exon and may serve as alternative transcriptional start sites (see Fig. 2A). Moreover, we observed differences in pulldown efficiencies, exposing a high enrichment for STAiRs 6 and 18, whereas STAiRs 1, 2, and 15 showed less efficient enrichment. In general, the oligo binding efficiencies depends on the accessibility of the target ncRNA, which might be covered by an interaction partner or be inaccessible due to RNA secondary structure.

Nuclear-cytoplasmic fractionation of INA-6 cells served to clarify the subcellular localization of STAiRs. For this purpose, primer pairs amplifying intronic regions were used to validate STAiR macroRNA expression (see Fig. 1A, red dots) while intron-spanning primer pairs for STAiRs 15 and 18 (see Fig. 1A, green bracket), were used to detect spliced transcripts. To determine the fractionation quality, MALAT1, a noncoding RNA essential for nuclear speckle formation^11^, was used as a nuclear control. A comparable nuclear distribution was observed for spliced STAiR15 (sSTAiR15; see Fig. 2B), confirming the already published nuclear localization of MIAT^12^. Therefore, STAiR15 is most likely identical to MIAT. Furthermore, STAiRs 1, 2, and 6, as well as the primary transcripts of STAiRs 15 and 18 (pSTAiRs 15 and 18) also showed a preferential nuclear enrichment, as expected for unprocessed RNAs. Moreover, the mature GAPDH and STAT3 mRNAs served as controls for cytoplasmic localization. Spliced STAiR18 (sSTAiR18) showed a distribution comparable to these mRNAs. In addition, we found that STAiR18 is polyadenylated (data not shown) and therefore conclude that STAiR18 is an mRNA-like ncRNA.

Except for spliced STAiR18, all STAiRs showed a preferential nuclear localization. We therefore analyzed whether STAiRs are chromatin-associated as it is published for many other long ncRNAs. We performed an RNA immunoprecipitation using antibodies targeting common histone modifications, like H3K4me3 and H3K36me3 representing active chromatin in promoters and genes, respectively. Moreover, H3K27me3 was included representative for heterochromatic regions. After histone pulldown, the enrichment of STAiRs and control RNAs was analyzed by RT-qPCR (see Fig. 2C). An association of RNAs with H3K36me3 cannot be taken as evidence for their epigenetic role as RNAs are necessarily produced from transcriptionally active loci and hence are expected to be in close proximity to this histone modification. Thus, H3K36me3 pulldown served rather as an internal control for transcriptional activity. Indeed, for all RNAs analyzed, except primary STAiR18 and HOTAIR, the specific H3K36me3-enrichment was highest, compared to the IgG control. Furthermore, the H3K4me3 pulldown for RNAs interacting with active promoter regions showed no significant enrichment of any tested RNA. In view of their enormous length, we used two different primer pairs for each STAiR to validate chromatin interactions. This resulted in comparable enrichments (see Supplemental Fig. 1). Interestingly, however, primary and to a certain extent, also spliced STAiR18 was found to be associated with H3K27me3, indicating a possible function in heterochromatin regulation. As a positive control for H3K27me3 interaction, HOTAIR, a long ncRNA described to recruit the Polycomb Repressive Complex 2 (PRC2) to genomic regions^13^ was used. Surprisingly the enrichment of primary STAiR18 by H2K27me3 pulldown was even higher than the enrichment of HOTAIR, indicating that unprocessed STAiR18 might be involved in chromatin silencing.

Taken together, STAiRs 1, 2, and 6 are rather unprocessed and preferentially nuclear macroRNAs, whereas STAiRs 15 and 18 correlate to already annotated lncRNAs MIAT and MIR4435-2HG, respectively, and are processed into smaller transcripts. Independent of its stage of procession, STAiR15 is located within the nucleus, which matches published data on MIAT. STAiR18 behaves like an mRNA, i.e. in its primary form it is located in the nucleus and after splicing gets transported to the cytoplasm. Furthermore, STAiR18 seems to be involved in epigenetic processes, as implied by its location at H3K27me3-marked heterochromatin.

### Expression studies expose STAiRs 1 and 2 as potential tumor markers for multiple myeloma

**Figure 3:**
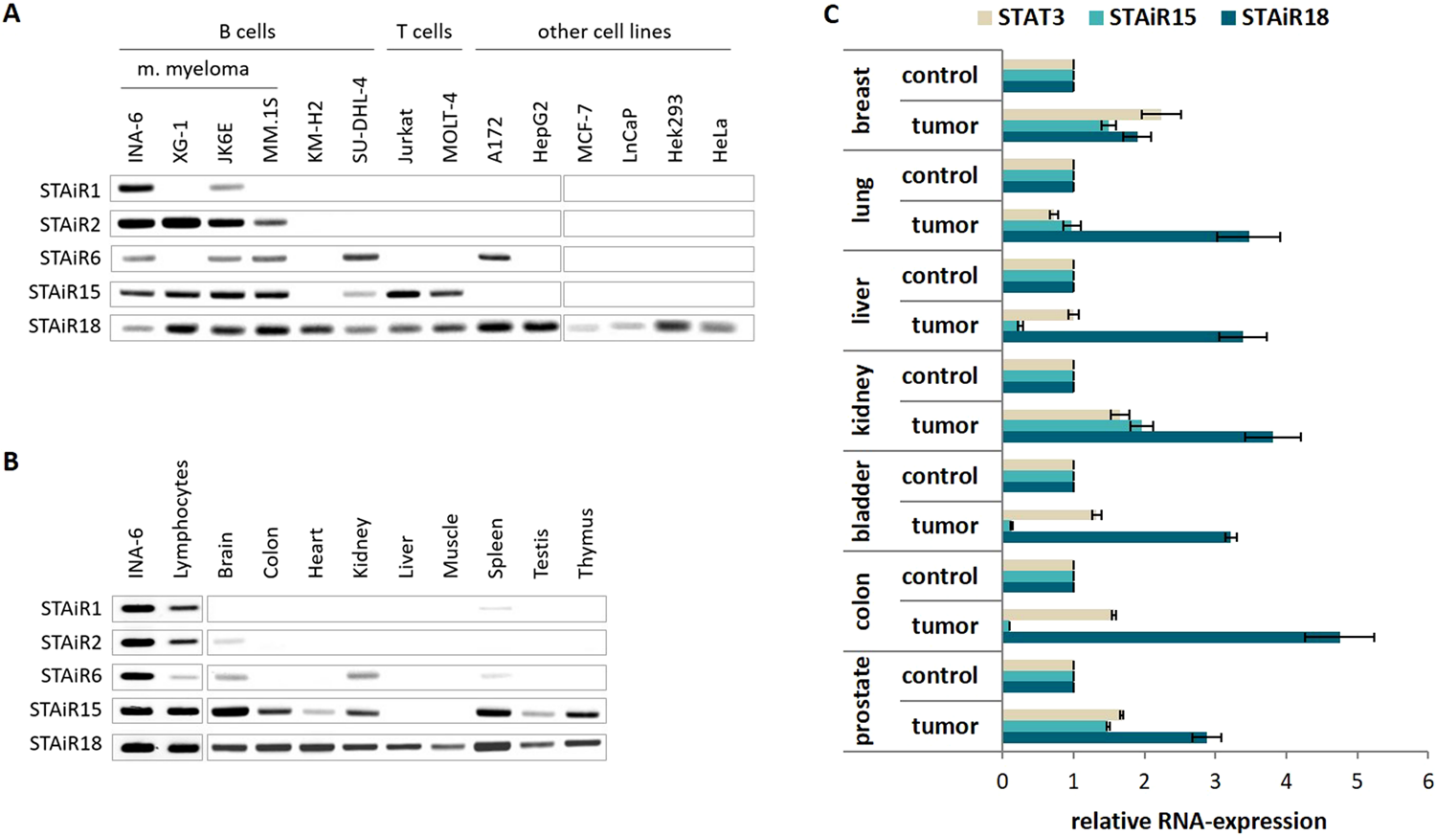
Tissue-specific expression of STAiRs. RNA was isolated from various (**A**) cell lines and (**B**) tissues. DNase-digested RNAs as well as Ambion’s FirstChoice® Human Tissue Total RNAs were reverse-transcribed and subjected to standard PCR using primer pairs for primary STAiRs, resulting in product sizes of 264 bp for STAiR1, 253 bp for STAiR2, 271 bp for STAiR6, 252 bp and 625 bp for STAiR15.1 and STAiR15.2, respectively, as well as 330 bp and 106 bp for STAiR18.1 and STAiR18.2, respectively. Individual gel runs are indicated by gray boxes. Raw data images are displayed in Supplemental Fig. 2. (**C**) Differential expression of spliced STAiRs 15 and 18 transcripts in tumorous and according healthy control samples. DNase-digested total RNA was reverse-transcribed and subjected to qPCR using specific intron-spanning primers for STAiRs 15, 18, and STAT3. Expression values were normalized to U6 RNA and compared to the corresponding healthy control sample. Data are expressed as mean ± SD (n ≥ 3). STAiR18 expression was significantly (p < 0.05) elevated in every tumor sample tested.

To determine the cell- and tissue-specific distribution of STAiR macroRNAs, we first analyzed their expression in various cell lines (Fig. 3A) and tissues (Fig. 3B). Expression of STAiRs 1 and 2 was restricted to multiple myeloma cells. These two STAiRs were not detected in any other cell line tested, suggesting their potential value as markers for multiple myeloma. STAiR6 expression was similarly restricted, with the exception of SU-DHL-4 follicular B-cell lymphoma and A172 glioblastoma cells. In contrast, STAiR15 was broadly expressed in lymphocytes and STAiR18 showed a ubiquitous expression. When organ-derived RNAs were analyzed (Fig. 3B), STAiR15 and STAiR18 depicted a broad expression in contrast to STAiRs 1, 2, and 6, which are barely detectable in healthy tissues. Here, the expression of STAiRs in INA-6 was used as a positive control to compare expression levels. Due to their broad occurrence, expression of STAiRs 15 and 18 was analyzed by a preliminary set of seven, mostly patient matched tumorous and healthy tissues (Fig. 3C and Supplemental Table 3). Here, intron-spanning primer pairs were used to detect the spliced transcripts. Given that STAT3 on the one hand induces the expression of STAiRs 15 and 18, and on the other itself is often overexpressed in various tumors^14^, it was used for comparison. We mostly observed an increased STAT3 expression in tumor samples compared to healthy counterparts except for liver and lung. However, based on the data, we cannot conclude a STAT3-dependent expression of STAiRs in this tumor entities. With low abundance in liver, bladder, and colon tumors, a moderate one in breast, lung, and prostate tumors and a high one in kidney tumors, sSTAiR15 showed a very tumor-specific expression. In contrast, after evaluating the limited collection of tumor samples, sSTAiR18 was found to be overexpressed in every tumor entity tested. Taken together, STAiRs 1 and 2 may serve as potential tumor markers for multiple myeloma, whereas STAiR15 is expressed differently in the analyzed tumors. STAiR18 is upregulated in all analyzed tumor tissues, indicating a potential function in tumorigenesis.

### Chromatin Isolation by RNA Purification demonstrates association of STAiRs with target RNAs

**Figure 4:**
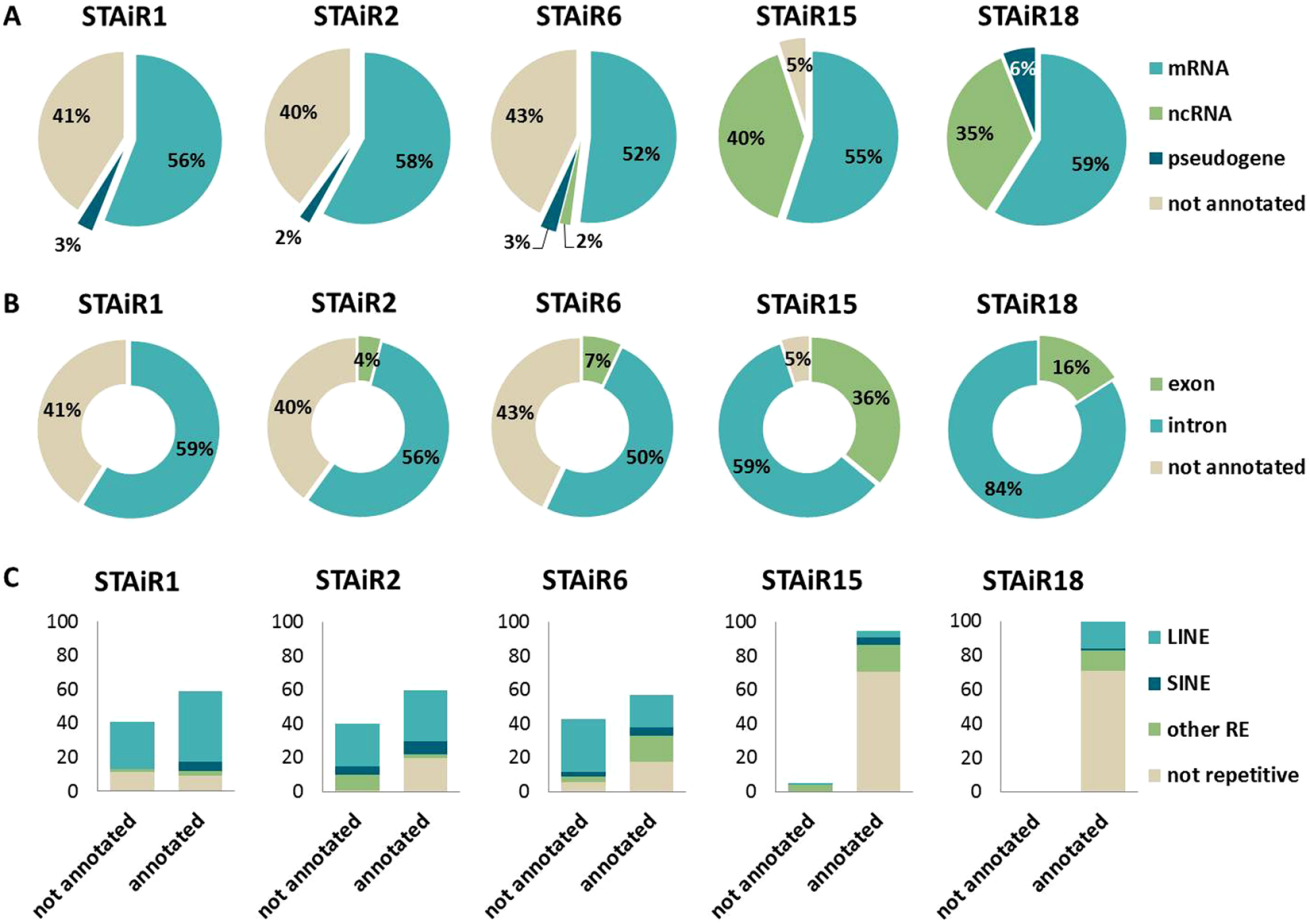
Identification of STAiR RNA-binding partners. ChIRP-RNA-sequencing was performed using 12 biotinylated oligonucleotides per STAiR RNA target (STAiRs 1, 2, 6, 15, 18) and 6 oligos for bacterial lacZ as a negative control. The pulldown was performed within crosslinked and chromatin-fragmented cell lysates of 2 × 10^7^ permanently IL-6 stimulated (10 ng/ml) INA-6 cells each. The RNA targets together with their RNA interaction partners were separated by streptavidin beads, following a total RNA preparation and DNase digestion. Library preparation (Scriptseq, Epicenter) and a subsequent NGS was performed for an RNA pool of five independent experiments. Identified reads were mapped to the human genome hg19. The reads belonging to the 100 most enriched genomic positions were analyzed for every STAiR pulldown compared to the lacZ negative control in order to identify (**A**) their origin within the human genome, (**B**) intragenic position, and (**C**) repetitive content and type.

As it is commonly published, certain lncRNAs display diverse functions in cancer pathways by both, interaction and regulation of different RNA species, predominately mRNAs^11, 15–17^. Thus, to identify RNA transcripts interacting with STAiRs, Chromatin-Isolation by RNA Purification (ChIRP) with a subsequent NGS of RNA was conducted. Therefore, cells were initially crosslinked to stabilize RNA-RNA-interactions, followed by sonication-caused cell lysis and fragmentation of chromatin. An oligo-based pulldown of STAiRs together with their binding partners was affiliated according to our previously performed RNA-CAPTURE-seq. The gained reads were mapped to the human genome and represent the targeted STAiRs as well as their RNA binding partners. With a fold change (FC) greater than five compared to the lacZ negative control, the 100 most enriched RNA subtypes for each STAiR were classified in Fig. 4A. For STAiRs 1, 2, and 6, a preferential interaction with not annotated RNA transcripts (40%) as well as mRNAs (50%) was observed. However, STAiRs 15 and 18 exhibited a preferred binding to annotated transcripts, of which 60% map to mRNAs and 40% map to ncRNAs. Apparently, different RNA subtypes were targeted by STAiRs.

Next we determined, whether the identified annotated RNAs comprise an exonic or intronic STAiR binding site (Fig. 4B). If an annotated transcript was targeted by STAiRs 1, 2 or 6, then it was located preferentially within an intron. STAiRs 15 and 18 showed little or no binding to not annotated transcripts. Here, the interaction also preferentially occured in intronic parts of the transcript (65–85%); however, exons were also bound to a certain extent. Given that mostly non-protein-coding parts of transcripts were directly bound by STAiRs, it should be clarified whether the binding site was located within repetitive elements. As illustrated in Fig. 4C, STAiRs 1, 2, and 6 preferentially targeted repetitive regions, like Long and Short Interspread Nuclear Elements (LINEs and SINEs), whereas STAiRs 15 and 18 did not.

Finally, to specify a potential binding motif, the software MEME (Multiple Em for Motif Elicitation) was used^18^. The top 25 interacting RNA transcripts enriched by the specific STAiR pulldown were analyzed. Those sequences in general depicted a length of about 50–200 nt. For STAiRs 1, 2, and 18, a significant motif of 40–50 nt in length was discovered, while STAiRs 6 and 15 showed no significant binding motifs (see Supplemental Fig. 3). For both, STAiRs 1 and 2, their most enriched RNA binding partners all harbored LINE elements within the binding site. Sequences bound by STAiR6 contained LINEs, SINEs and other repetitive elements. However, due to their elevated length and weak significance the found binding motifs appear of questionable reliability. Taken together, STAiRs seem to divide into two classes of long ncRNAs, first the rather unprocessed and myeloma specific macroRNAs (STAiRs 1, 2 and 6) and second the mRNA-like transcripts (STAiRs 15 and 18) by means of their binding preferences.

### IL-6 stimulation induced alternative splicing at the DCC locus

**Figure 5:**
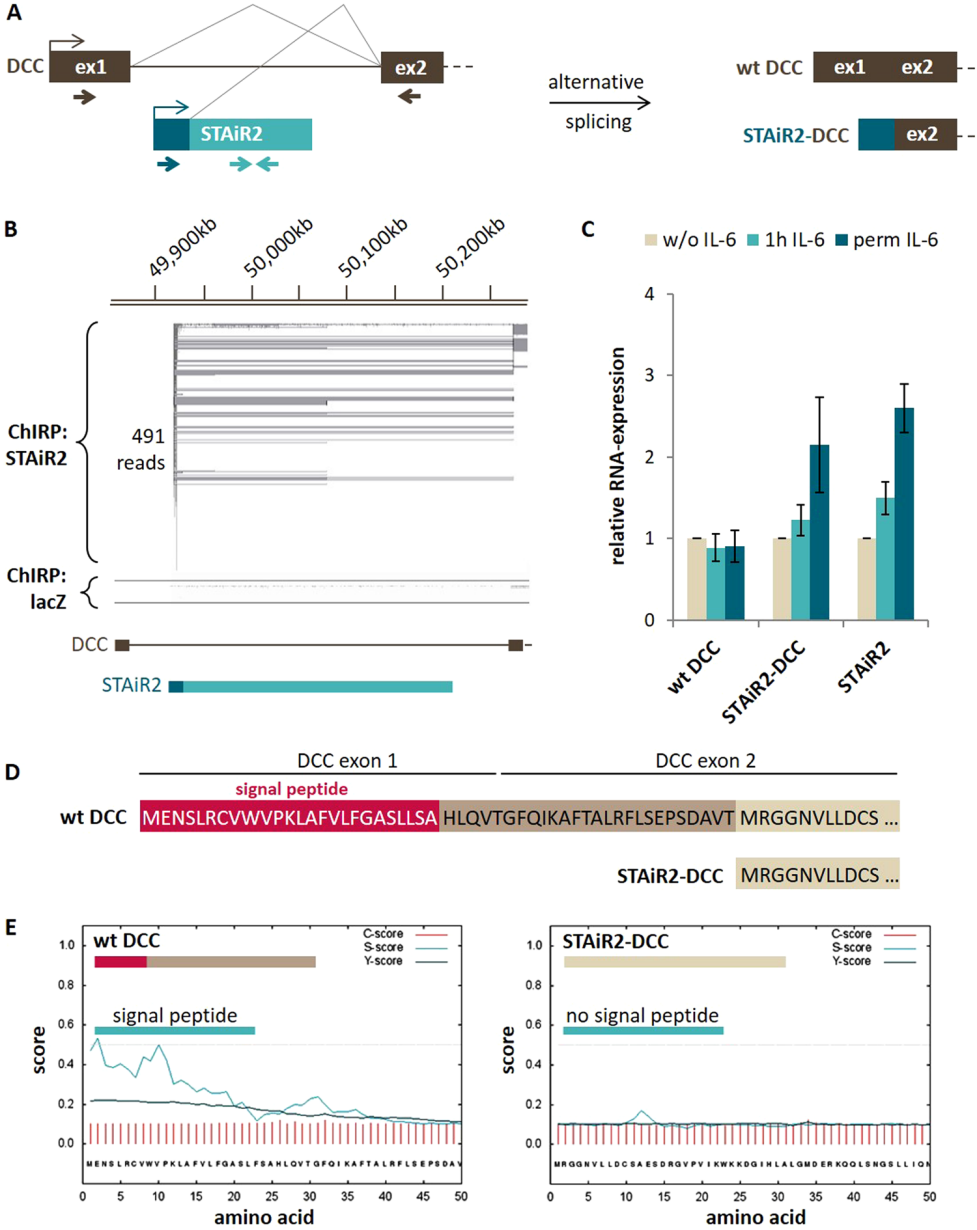
Alternative splicing of DCC and STAiR2 leads to an impaired DCC function. (**A**) Scheme of the alternative splicing event connecting the 5′-region of STAiR2 with DCC exon2. Primer pairs used for variant validation are indicated at the bottom (brown for wild type DCC, dark blue (forward) and brown (reverse) for STAiR2-DCC hybrid, and light blue for STAiR2). (**B**) ChIRP-seq data of STAiR2 confirming a splice event from STAiR2 5′-region and DCC exon2. STAiR2-ChIRP-seq was performed as described in Fig. 4. Gained reads for the STAiR2 locus were visualized by IGV compared to reads detected after lacZ negative control. The location of DCC host gene and STAiR2 ncRNA are shown at the bottom. (**C**) Validation of STAiR2-DCC hybrid. DNase-digested total RNA of withdrawn, 1 h IL-6 restimulated and permanently IL-6 treated INA-6 cells was reverse-transcribed and subjected to qPCR using specific primers shown in Fig. 5A. Expression values were normalized to U6 RNA and compared to the unstimulated control sample. Data are expressed as mean ± SD (n ≥ 3). (**D**) In frame open reading frames of the N-terminal regions of wild type DCC and STAiR2-DCC hybrid analyzed by NCBI ORF Finder. STAiR2-DCC hybrid depicts a downstream translational start site and lacks the signal peptide sequence (red) exclusively present in wild type DCC. (**E**) Analysis of the first 50 amino acids of wild type DCC (left) and STAiR2-DCC hybrid (right) by SignalP-4.1, a software to check for signal peptides. The C-score (red lines) detects signal peptide cleavage sites, the S-score (light blue line) detects the signal peptide position and the Y-score (dark blue line) is the geometric average of both scores.

STAiR2 is expressed from the first intron of the protein-coding DCC gene, short for Deleted in Colorectal Cancer (see Fig. 5A compared to Fig. 1A), encoding a transmembrane receptor that operates as a tumor suppressor^19, 20^. Therefore, we aimed at elucidating whether STAiR2 has an impact on DCC expression in multiple myeloma cells. Using our ChIRP-seq data, we found a splice event connecting the 5′-region of STAiR2 with the second exon of DCC (Fig. 5A and B), creating a not yet annotated first alternative exon of DCC generated by alternative splicing. To verify this alternative DCC exon with STAiR2 origin, different primer pairs were designed (see Fig. 5A) to specifically amplify wild type DCC, STAiR2, and STAiR2-DCC hybrids by qPCR. Our data show that upon IL-6 stimulation both, STAiR2 as well as the STAiR2-DCC hybrid were induced in INA-6 cells, whereas the annotated DCC mRNA depicted no relevant expression (Fig. 5C). However, by comparing RNA expression levels with STAT3 (100%), the mRNA expression of wild type DCC is with 5% rather low, compared to 51% STAiR2 expression (data not shown). Moreover, after coprecipitation of H3K36me3-associated RNAs by RIP, DCC mRNA was less enriched than STAiR2 (see Supplemental Fig. 1), indicating that the expression of DCC wild type plays a minor role in INA-6 myeloma cells compared to both, STAiR2 and the hybrid.

Last, we tested whether alternative splicing resulted in changes of the DCC open reading frame. Therefore, we checked the nucleotide sequence of the potential STAiR2-DCC hybrid and compared it with wild type DCC by NCBI ORF Finder. The translational start site of wild type DCC is located within the first exon. Due to the connection of STAiR2 5′-region with DCC exon 2, a more distal translational start site in DCC exon 2 is used, which leads to a loss of the first 51 amino acids (Fig. 5D). Furthermore, the nucleotide sequence surrounding the translational start site of alternatively spliced STAiR2-DCC does not contain a Kozak sequence, indicating that the fusion transcript is unlikely to be translated into a functional protein. Additionally, the wild type DCC protein contains a hydrophobic signal peptide at the N-terminus, which determines its secretory fate and further enables its transmembrane anchoring^21^. The shifted translational start of STAiR2-DCC hybrid would lead to a complete loss of signal peptide, which was confirmed by analyses of the first 50 amino acids of both, wild type and hybrid DCC by SignalP-4.1 software (Fig. 5E).

Taken together, intragenic expressed STAiR2 regulates its tumor suppressor host gene *DCC* by alternative splicing. However, due to the lack of Kozak sequence and a signal peptide, the STAiR2-DCC fusion transcript is very unlikely to form a functional protein. This hypothesis was further supported by Western blot (shown in Supplemental Fig. 4), depicting no STAiR2-DCC protein product. Hence, the IL-6-induced alternative splicing of the DCC locus probably leads to an impaired protein function and may contribute to myeloma cell survival.

## Discussion

Various lncRNAs have been described as highly tissue-specific drivers for cancer phenotypes^16^. For multiple myeloma, several microRNAs contribute to this hematological malignancy^22^, like for example the oncogenic and anti-apoptotic miR21^7, 8^. However, a characterization of long noncoding RNAs in multiple myeloma is still lacking. In this study, we introduce STAiRs, five IL-6/STAT3-induced long ncRNAs in INA-6 multiple myeloma cells. Except for STAiR2, which is encoded within the first intron of the tumor suppressor gene *DCC*, STAiRs 1, 6, 15, and 18 are expressed from intergenic regions. For the novel, unannotated transcripts of STAiRs 1, 2, and 6, we did not obtain any evidence for splicing by CAPTURE-RNA-seq. For those STAiRs, read coverage peaked in the areas targeted by CAPTURE oligonucleotides while it decreased in areas outside the target region. We do not interpret this as an indication of splicing as the analysis of the sequencing data did not yield any split reads over splice junctions and the length of the transcripts might cause breaking of these long RNA molecules during sample processing. Furthermore, preliminary data indicate the existence of STAiR macroRNA transcripts by Northern blot analysis. The general variation of STAiR pulldown efficiencies may be explained by transcript accessibility. For example, by protein occupation and RNA secondary structure, some oligo binding sites within the target sequence might be blocked and impair the oligo-based-pulldown. In addition, STAiRs 1, 2, and 6 are macroRNAs and preferentially retained in the cell nucleus. Due to their exclusive expression in multiple myeloma cell lines, STAiRs 1, 2, and possibly 6 may serve as tumor markers. This is supported by our preliminary data on myeloma patient samples, showing expression of STAiRs 1, 2 and 6 specifically in myeloma samples compared to healthy controls.

To date, it is widely understood that lncRNAs could identify cellular pathologies such as cancer, or even provide prognostic values^16^. The lncRNAs H19 and PCA3 for example serve as biomarkers for gastric and prostate cancer, respectively^23, 24^, and overexpression of HOTAIR in breast cancer is correlated with metastasis potential and poor prognosis^25^.

In contrast, the more broadly expressed STAiR15 and the ubiquitous STAiR18 are frequently spliced and match to the already annotated ncRNA genes *MIAT* and *MIR4435-2HG*, respectively. MIAT is known to be differentially expressed in cardiovascular diseases and mental disorders^26^. Here we observed elevated levels of STAiR15 also in breast and kidney cancer samples whereas its expression was strongly reduced in bladder, liver, and colon cancer, indicating a very tissue- and tumor-specific expression pattern. Furthermore, we observed the published nuclear enrichment of MIAT/STAiR15^10^ in myeloma cells as well. In contrast, STAiR18 was found in both, nucleus and cytoplasm. The primary transcript preferentially remains in the nucleus whereas the spliced transcript is exported into the cytoplasm. We showed that, while in the nucleus, STAiR18 interacts with trimethylated H3K27, which is known as a mark for silenced chromatin. Given that RNAs are transcribed from open and active chromatin, STAiR18 might be involved in silencing transcription by acting on heterochromatic regions within the genome in *trans*, like HOTAIR^13, 25, 27^. Furthermore, STAiR18 was overexpressed in all tumors tested compared to their healthy controls. This indicates a potential function in tumorigenesis. However, the molecular function of STAiR18/MIR4435-2HG still remains unknown. Like STAiR18, the lncRNA ANRIL also depicts an increased expression in various cancer types. ANRIL is transcribed from the p15/CDKN2B, p16/CDKN2A, p14/ARF cluster^28^ and regulates gene expression in *trans*, affects cell adhesion, proliferation, and apoptosis by binding to PRC1 and PRC2 proteins and recruiting them to promoters of target genes^29^. Hence, STAiR18 might be involved in similar chromatin modulating processes. To clarify this, further investigations of STAiR18 protein interaction partners are needed.

Some lncRNAs that are known to play an important role in cancer pathways have been shown to target and influence other cellular RNAs^16^. The nuclear lncRNA MALAT1 for example is involved in alternative splicing by interacting with splicing factors and pre-mRNAs^11^, whereas lincRNA-p21 binds the JUNB mRNA and impairs its translation^17^. Additionally, lncRNAs are involved in Staufen-1-mediated mRNA-decay through interaction with 3′-UTRs of mRNAs^15^. In contrast, together with lncRNA TINCER, Staufen-1 stabilizes mRNAs. LncRNAs bind Staufen-1 through Alu elements, which are also required in the targeted mRNA. Moreover, gene regulation caused by ANRIL depends on Alu motifs as well, which are found in both, the promoters of ANRIL target genes and the ANRIL RNA transcript itself^29^. Thus, we analyzed STAiR-RNA interactions and whether these depend on repetitive sequences by ChIRP-seq. We observed that about one half of the RNAs targeted by STAiRs are mRNAs, while the other half was represented by either unannotated or annotated ncRNA transcripts for STAiRs 1, 2, and 6 or for STAiRs 15 and 18, respectively. This discrimination of STAiRs 1, 2, and 6 from STAiRs 15 and 18 was further observed by the proportion of repetitive elements within the targeted RNA. STAiRs 1, 2, and 6 preferentially bind RNAs containing long and short interspersed elements (LINEs and SINEs) or other repetitive sequences. In contrast, the RNAs targeted by STAiRs 15 and 18 consist predominantly of nonrepetitive sequences. Nucleotide sequence analysis of the top STAiR-enriched RNAs yielded no reliable shared motif. Hence, we argue that binding of STAiRs to their specific RNA subtypes is mediated rather by a connecting protein or complex secondary structures than a specific sequence pattern. Therefore, a detailed investigation of proteomic STAiR binding partners by ChIRP with a subsequent mass spectrometry analysis is needed for a better understanding of their molecular function.

Exemplarily for STAiR2, we began to characterize the operating mechanisms of STAiRs. As mentioned before, STAiR2 is expressed from the first intron of the *DCC* gene, which encodes a transmembrane receptor for netrin-1. In the absence of netrin-1, DCC induces apoptosis^30^. Given that its inactivation is associated with multiple tumor types^19, 20^, DCC is currently believed to be a conditional tumor suppressor^31^. After translation, the signal peptide of DCC located at the N-terminus determines both, the release of DCC from the endoplasmic reticulum (ER) and its anchoring within the cell membrane^32^. DCC is described as being alternatively spliced, generating two isoforms that diverge in their extracellular domain^33^, however, both variants are not affected by STAiR2 expression in INA-6 cells. Here, we introduce another DCC isoform, arising from a splice event that connects the 5′-region of STAiR2 with the second exon of DCC after IL-6 stimulation in INA-6 cells. This particular isoform was also described by Nagoshi *et al*. in multiple myeloma previously^34^, however without knowing that there is a long ncRNA involved. Consequentially, the translational start site of STAiR2-DCC hybrid is shifted from DCC exon1 to exon2 and results in a truncated DCC N-terminus, a loss of Kozak sequence and signal peptide. Therefore, the STAiR2-DCC protein product may neither be translated, nor integrated into the membrane and hence, cannot execute its normal function. In accordance with Nagoshi *et al*., this hypothesis was further supported by Western blot analyses, only showing expression of the wild type but not the STAiR2-DCC fusion protein. By means of STAiR2 we could reveal, that macroRNAs are able to modulate tumor suppressor functions of protein-coding genes in *cis* and thereby facilitate pathological processes, like tumorigenesis.

Taken together, we discovered and described five lncRNAs, termed STAiRs, which are induced by IL-6-activated STAT3 in INA-6 multiple myeloma cells. An analysis of transcript processing, intracellular localization, chromatin interaction and tumor-specific expression exposed different properties leading to a possible classification into two STAiR groups, each with distinct characteristics. Group one includes the rather unprocessed and nuclear retained STAiR macroRNAs 1, 2, and 6, which show a myeloma-specific expression and therefore might serve as suitable biomarkers. Moreover, STAiRs 1, 2 and 6 may execute specific functions in myeloma tumor integrity, as it was indicated exemplarily by STAiR2 and its ability to inhibit the tumor suppressor function of DCC in INA-6 cells. The other group comprises multiple-spliced and ubiquitously expressed STAiRs 15 and 18, of which STAiR18 seems to be involved in chromatin silencing and, by being overexpressed in multiple tumors, seems to globally influence tumor establishment or maintenance. Thus, STAiR18 is a promising candidate for further investigations in regard to determine its detailed molecular function.

## Methods

### Cell culture cells and tissue samples

The human multiple myeloma cell lines INA-6 and JK-6E were generously provided by the Gramatzki group (Kiel, Germany)^35^. XG-1 cells were supplied by Bernard Klein (Montpellier, France). Other human myeloma, leukemia, and lymphoma cell lines U266, JK-6E, Jurkat, KM-H2, MM1S, MOLT-4, and SU-DHL-4 were obtained by ATCC or DSMZ and maintained in RPMI1640 + GlutaMAX™ (LIFE Technologies, Carlsbad, California, USA), supplemented with 10% fetal calf serum (Lonza, Basel, Switzerland) and 1% penicillin/streptomycin (LIFE Technologies). For INA-6 and JK-6E cells, 1 ng and for XG-1 2 ng IL-6 were added per ml medium. Where indicated, INA-6 cells were withdrawn from IL-6 for at least 12 h with an optional IL-6 restimulation. The other adherent cell lines (A172, MCF-7, HEK293, HepG2, LnCAP, and HeLa, all obtained by ATCC or DSMZ) were cultured in DMEM + GlutaMAX™ (LIFE Technologies). For MCF-7 cells, cell culture medium was additionally supplemented with 1% sodium pyruvate (LIFE Technologies) and 1% MEM non-essential amino acids (LIFE Technologies). Identity authentication was performed either morphologically under the microscope or by typical target gene patterns in response to stimulation. Every batch was tested negative for mycoplasma contamination. Additionally, the FirstChoice® Human Total RNA Survey Panel (LIFE Technologies) as well as RNAs from human lymphocytes (generously provided by the group of Prof. S. Hauschildt, Leipzig, Germany), tumorous and healthy tissues (amsbio/BioChain; prostate normal: R1234201-50; prostate tumor: CR560070; bladder normal: R1234010-50; bladder tumor: R1235010-10; breast tumor and control: R8235086-PP-10; colon tumor and control: R8235090-PP-10; kidney tumor and control: R8235142-PP-10; liver tumor and control: R8235149-PP-10; lung tumor and control: R8235152-PP-10) were used.

### Nuclear-cytoplasmic fractionation

Cellular fractionation was performed by adding cytoplasmic buffer (20 mM Hepes pH 7.4, 10 mM KCl, 1 mM EDTA, 0.1 mM NaVO_4_, 10% Glycerin, 1 mM DTT, 0.1 mM PMSF, 1 × complete protease inhibitor (Roche Diagnostics, Rotkreuz, Switzerland), 0.2% NP-40) to cell pellets. After incubation for 5 min on ice and centrifugation, supernatants served as cytoplasmic fractions, whereas pellets were resuspended in equal volumes of nuclear buffer (same composition except 420 mM KCl and 20% glycerin) to receive the nuclear fraction after 30 min incubation on ice and centrifugation. Each nuclear-cytoplasmic fractionation was performed in a minimum of three independent biological replicates. Data are shown as means, and error bars represent standard deviation (SD).

### Isolation of RNA, cDNA synthesis and analysis

RNA was isolated with TRIzol (LIFE Technologies), following the manufacturer’s protocol. RNA was DNase-digested using TURBO-DNA-free kit (LIFE Technologies). Reverse transcription of RNA was conducted using the RevertAid First Strand cDNA synthesis kit (Thermo, Waltham, Massachusetts, USA). Analysis of cDNA was performed using either Light Cycler® Fast Start DNA Master Plus SYBR Green kit (Roche) as described by the manufacturer using Light Cycler® or by standard PCR. Primers are listed in the Supplemental Table 1. Each (RT)-qPCR reaction was performed in at least three independent biological replicates. Data are shown as means and error bars given as standard deviations (SD). For indicated experiments, a two-sided t-test was used to assess statistical significance.

### RNA interference

Cells were transfected with 200 pmoles StealthTM siRNA targeting STAT3 exon 9 (5′-TCTCAACTTCAGACCCGTCAACAAA-3′) per 5 × 10^6^ cells. A medium GC negative control (Invitrogen, Waltham, Massachusetts, USA) was used for subsequent normalization. Transfection was carried out using the NEON™-Kit and the microporator MP100 Digitalbio (LIFE Technologies) according to the manufacturer’s instructions. Three pulses of 1600 V and 10 ms were applied.

### Tiling array analysis

The tiling array procedure and data analysis was performed as described in Hackermüller *et al*.^9^ using TileShuffle^36^. The RNA expression studies are accessible from the Gene Expression Omnibus (GEO) database. Details regarding the transcriptional activity in response to STAT3 activation is available through [GEO:GSE44657] for INA-6 cells deprived from IL-6 for 12 h, [GEO:GSE44656] for 1 h restimulated cells, and [GEO:GSE44658] for cells permanently cultured in IL-6. Significant changes of expression are stored in [GEO:GSE44659].

### Chromatin isolation by RNA purification

Chromatin isolation by RNA purification (ChIRP) was conducted according to Chu *et al*.^37^. In short, 3′-biotinylated oligonucleotides (listed in the Supplemental Table 2) were designed complementary to the target ncRNA (STAiRs 1, 2, 6, 15, and 18) and to bacterial lacZ RNA as a negative control. 2 × 10^7^ cells per pulldown were fixed using 1% formaldehyde for 10 min and sonicated for 20 min using Bioruptor® (Diagenode, Seraing, Belgium). Pulldown was done by adding 100 pmol oligo mixture (12 oligos targeting each STAiR in different positions) per 1 ml cell lysate, followed by an immobilization to streptavidin T1 magnetic Dynabeads® (Thermo). RNA pulldown fractions were analyzed by NGS.

### CAPTURE RNA-sequencing

Total RNA was prepared from 2 × 10^7^ cells per pulldown and incubated with corresponding ChIRP oligonucleotides without prior crosslinking or sonication. The subsequent experimental procedure was performed according to the ChIRP protocol. RNA pulldown fractions were analyzed by NGS.

### Next-generation sequencing (NGS)

Next generation RNA sequencing was performed using the scriptseq™ v2 RNA-seq Library Preparation kit (Epicentre, Madison, Wisconsin, USA) as described by the manufacturers. For each sequencing reaction, four independent biological replicates of an experiment were pooled before library preparation. After successful library preparation, the sequencing reactions was conducted by MiSeq™(Illumina). For analysis, adapters were removed using Cutadapt^38^, followed by a mapping to the human genome hg19 using TopHat^39^. Data were visualized via UCSC Genome Browser^40^ or Integrative genomics viewer^41^. Raw and processed data sets of STAiR CAPTURE- and ChIRP-seqs are stored in the GEO database on platform GPL15520 (Illumina Miseq). The CAPTURE-seqs of STAiR1, STAiR2, STAiR6 and their corresponding lacZ negative control are stored in [GEO:GSM2496671], [GEO:GSM2496672], [GEO:GSM2496673], and [GEO:GSM2496677], respectively. CAPTURE-seqs of STAiR15, STAiR18 and lacZ are stored in [GEO:GSM2496674], [GEO:GSM2496675], and [GEO:GSM2496676], respectively. ChIRP-seqs of STAiR1, STAiR2, STAiR6 and lacZ are stored in [GEO:GSM2496678], [GEO:GSM2496679], [GEO:GSM2496680], and [GEO:GSM2496684], respectively. ChIRP-seqs of STAiR15, STAiR18 and lacZ are stored in [GEO:GSM2496681], [GEO:GSM2496682], and [GEO:GSM2496683], respectively.

### RNA immunoprecipitation

RNA immunoprecipitation (RIP) was performed using the EZ ChIP kit (Upstate, Lake Placid, New York, USA) according to the manufacturer’s instructions together with antibodies targeting H3K4me3, H3K27me3 (Cell Signaling, Cambridge, UK; #9727S and #9733S, respectively), H3K36me3 (Abcam, Cambridge, UK; ab9050), and an IgG negative control (Abcam; ab37415). For each IP approach 5 µg antibody were used for 5 × 10^6^ cells. SUPERase In™ (Thermo) was added to all buffers and the duration of crosslink reversal amounts to 2 h.

### Data availability statement

The RNA expression studies (Tiling Arrays) are accessible from the Gene Expression Omnibus (GEO) database. Details regarding the transcriptional activity in response to STAT3 activation is available through [GEO:GSE44657] for INA-6 cells deprived from IL-6 for 12 h, [GEO:GSE44656] for 1 h restimulated cells, and [GEO:GSE44658] for cells permanently cultured in IL-6. Significant changes of expression are stored in [GEO:GSE44659].

The studies regarding STAiR transcript architecture determined by CAPTURE-seq and STAiR RNA interaction partners determined by ChIRP-seq are also accessible from the GEO database on platform GPL15520 (Illumina Miseq). CAPTURE-seqs of STAiR1, STAiR2, STAiR6 and their corresponding lacZ negative control are stored in [GEO:GSM2496671], [GEO:GSM2496672], [GEO:GSM2496673], and [GEO:GSM2496677], respectively. CAPTURE-seqs of STAiR15, STAiR18 and their corresponding lacZ negative control are stored in [GEO:GSM2496674], [GEO:GSM2496675], and [GEO:GSM2496676], respectively. ChIRP-seqs of STAiR1, STAiR2, STAiR6 and their corresponding lacZ negative control are stored in [GEO:GSM2496678], [GEO:GSM2496679], [GEO:GSM2496680], and [GEO:GSM2496684], respectively. ChIRP-seqs of STAiR15, STAiR18 and their corresponding lacZ negative control are stored in [GEO:GSM2496681], [GEO:GSM2496682], and [GEO:GSM2496683], respectively.

## Electronic supplementary material

Supplementary Info
