# Supplementary material for: STAT3-induced long noncoding RNAs in multiple myeloma cells display different properties in cancer
Source: Sci Rep. 2017 Aug 11;7:7976. doi: 10.1038/s41598-017-08348-5 (PMC5554185; doi:10.1038/s41598-017-08348-5)
Supplement: Supplementary file 1 — Supplementary Info [file 41598_2017_8348_MOESM1_ESM.pdf]

STAT3-induced long noncoding RNAs in multiple myeloma cells display different properties in cancer

Supplementary information

Stefanie Binder[1,2,7,¶*], Nadine Hösler[1,2,7,¶], Diana Riedel[1,2], Ivonne Zipfel[1,2], Tilo Buschmann[2,3], Christoph Kämpf[2,4,6,7], Kristin Reiche[2,3,4], Renate Burger[8], Martin Gramatzki[8], Jörg Hackermüller[2,4,5], Peter F. Stadler[2,6,7,9-14] and Friedemann Horn[1-3,7]

Supplemental Table 1: (q) PCR primers

Supplemental Table 1: Listing of used (q) PCR primers complementary to the Methods section of the manuscript.

The following PCR primers were designed using Primer3 software, blasted against the human genome hg19 and provided by Eurofins Scientific, Luxembourg, Luxembourg.

Primer	Sequence	Comment	Product size
STAiR1.1_for<br>STAiR1.1_rev	5'-CTCAGTTTGGCATCCGTTTT-3'<br>5'-ATTGACTTCCCAGGCCTTTT-3'	Primary transcript, macroRNA	264bp
STAiR1.2_for<br>STAiR1.2_rev	5'-GCTGTGATGGACAGAAGCAA-3'<br>5'-TGAACACAGCAAGCACATGA-3'	Primary transcript, macroRNA	104bp
STAiR2.1_for<br>STAiR2.1_rev	5'-TCGGTATGTGGGTTTGGTTT-3'<br>5'-CTGGCTTCTCAGCTGGACTT-3'	Primary transcript, macroRNA	253bp
STAiR2_5´exon_for	5'-ATTTCCCGTTCCAAGTCCCA-3'	For detection of STAiR2-DCC hybrid	
DCC_for<br>DCC_rev	5'-CAGCGCGCATCTTCAAGTAA-3'<br>5'-GACATTTCCTCCCCGCATTG -3'	Spliced transcript, intron-spanning	
STAiR6.1_for<br>STAiR6.1_rev	5'-ATCTCATCGGGTCAGTGGAC-3'<br>5'-CTAATCCAGCCTGCTCCTTG-3'	Primary transcript, macroRNA	271bp
STAiR6.2_for<br>STAiR6.2_rev	5'-GGCTAGGCTGAGGAAGGTCT-3'<br>5'-CTGCAAGTGGCATTACGAGA-3'	Primary transcript, macroRNA	280bp
STAiR15.1_for<br>STAiR15.1_rev	5'-TCTGATGACCCTCTGGGTTC-3'<br>5'-GTGCTTTAAACGGGTGCATT-3'	Primary transcript, macroRNA	252bp
STAiR15.2_for<br>STAiR15.2_rev	5'-GCTGCCTACAGAAAAGAAAGCTG-3'<br>5'-CAGTGTTGCATGGTGTGATTAAC-3'	Primary transcript, macroRNA	625bp
MIAT_for = sSTAiR15_for<br>MIAT_rev = sSTAiR15_rev	5'-GGCTCCTGGTCCTTCTTTCT-3'<br>5'-TGACCGCTAAGTCACTGTGG-3'	Spliced transcript, intron-spanning	
STAiR18.1_for<br>STAiR18.1 _rev	5'-TGGCTGAGTTGTGGACAGAG-3'<br>5'-CCCCCAGTACTGCAGGTAAA-3'	Primary transcript, macroRNA	330bp
STAiR18.2_for<br>STAiR18.2_rev	5'-TGAGAATACATATGTGTGCAAGGA-3'<br>5'-GGAACACTCTGAAAAACACCAA-3'	Primary transcript, macroRNA	106bp
sSTAiR18_for<br>sSTAiR18_rev	5'-CGTGCCTGTCTTCAGATCTTC-3'<br>5'-GGGAATCTTTCAGCTGCATT-3'	Spliced transcript, intron-spanning	
MALAT1_for<br>MALAT1_rev	5'-CTACTGGGCTGACATTAACT-3'<br>5'-ACAAAAGCTACCATCAGAAG-3'	Primary and spliced transcript	
GAPDH_for<br>GAPDH_rev	5'-GTCAGTGGTGGACCTGACCT-3'<br>5'-AGGGGAGATTCAGTGTGGTG-3'	Spliced transcript, intron-spanning	
HOTAIR_for<br>HOTAIR_rev	5´-GGGAGTTCCACAGACCAACA-3´<br>5´-TTAGGGACCTGAGGGTCTAAGTC-3´	Primary transcript	
U6_for<br>U6_rev	5'-CTCGCTTCGGCAGCACA-3'<br>5'-AACGCTTCACGAATTTGCGT-3'	Primary transcript	
STAT3_for<br>STAT3_rev	5'-GAGGGAACAAGCCCCAAC-3'<br>5'-CTTGCAGGAAGCGGCTATAC-3'	Spliced transcript, intron-spanning	
SGK1_for<br>SGK1_rev	5'-TTCTATGCTGCTGAAATAGCCA-3'<br>5'-CCAGTCCACAGTCCTGTCATAA-3'	Spliced transcript, intron-spanning	
Pri-miR21_for<br>Pri-miR21_rev	5'-CATTGTGGGTTTTGAAAAGGTTA-3'<br>5'-CCACGACTAGAGGCTGACTTAGA-3'	Primary transcript	
Mature-miR21_RT primer	5'-<br>GTCGTATCCAGTGCAGGGTCCGAGGTATTC<br>GCACTG -3'	Stemloop RT primer	

Mature-miR21_for	5'-GCCCGCTAGCTTATCAGACTGATG-3'	Mature miR21
Mature-miR21_rev	5'-GTGCAGGGTCCGAGGT-3'	

Supplemental Table 2: ChIRP oligonucleotides

Supplemental Table 2: Listing of used ChIRP oligonucleotides complementary to the Methods section of the manuscript. The following 3´-biotinylated DNA oligonucleotides (= BITEG; with a TEG spacer between the oligo and biotin, non-overlapping, 20-25mers, blasted against the human genome hg19) were designed using www.singlemoleculefish.com and provided by Eurofins Scientific, Luxembourg, Luxembourg.

Oligo	Sequence
lacZ1	5'-CCAGCTTTCATCAACATTAAATGTG-3'-BITEG
lacZ2	5'-ATAAAGAAACTGTTACCCGTAGGTA-3'-BITEG
lacZ3	5'-CGTTAAAGTTGTTCTGCTTCATCAG-3'-BITEG
lacZ4	5'-GCCGTTTTCATCATATTTAATCAGC-3'-BITEG
lacZ5	5'-AGTTCAATCAACTGTTTACCTTGTG-3'-BITEG
lacZ6	5'-TGATAAATAAGGTTTTCCCCTGATG-3'-BITEG
STAiR1.1	5'-AACTGGAGTCTCAGCCAAAG-3'-BITEG
STAiR1.2	5'- TCTCTTAAGTGGGTTTCAGT -3'-BITEG
STAiR1.3	5'-AGAAGGCTTTTCAGAGATCT-3'-BITEG
STAiR1.4	5'- AGTGCCAAGAAGTAAGGGTA -3'-BITEG
STAiR1.5	5'-GTCCACATTCAAACTTTACA-3'-BITEG
STAiR1.6	5'- CAGTAATCCGTTTAACAGCA -3'-BITEG
STAiR1.7	5'-ATCCCAACTCACTCCATTTC-3'-BITEG
STAiR1.8	5'- AGACTTACTTGGTCTTCTGG -3'-BITEG
STAiR1.9	5'-GTCAGGAAGGGAAATCAGTT-3'-BITEG
STAiR1.10	5'- GCATCAACTGTGCTCAGAAC -3'-BITEG
STAiR1.11	5'-AGCCGATCCAAGGTAATATT-3'-BITEG
STAiR1.12	5'- TCCCTTGTCATGTTTAAAGG -3'-BITEG
STAiR2.1	5'- CTTTCTCAAAGTGAGTTGCC -3'-BITEG
STAiR2.2	5'- TTGTGACCATAATTGCTGGG -3'-BITEG
STAiR2.3	5'- CTGTGTCACTCCAACTGATA -3'-BITEG
STAiR2.4	5' -GTTGTGGAATAGGTTGAGCT -3'-BITEG
STAiR2.5	5'- AGTTTGCCTGGAATCATACA -3'-BITEG
STAiR2.6	5'- GGACTAAATCACAGGGCGAC -3'-BITEG
STAiR2.7	5'- CCCATCCGTTATGAATCAAC -3'-BITEG
STAiR2.8	5'- CTAGGGAAGATTTCACCTGT -3'-BITEG
STAiR2.9	5'- GGTAGCACTCTAATGGCAAT -3'-BITEG
STAiR2.10	5'- GTCACAGAGTCAATACCACT -3'-BITEG
STAiR2.11	5'- ATAGCAATTGGTGCTACTGC -3'-BITEG

STAiR2.12	5'- CAGCATCAATTTTGGTGCAC -3'-BITEG
STAiR6.1	5'- CTGACACTAAGGGAGGCATA -3'-BITEG
STAiR6.2	5'- AGTTGGGAAACCTCTTCGTC -3'-BITEG
STAiR6.3	5'- CATCTCCGGCATTATGACAC -3'-BITEG
STAiR6.4	5'- CTTGGGTACGAGGAGGTTAA -3'-BITEG
STAiR6.5	5'- GAGAGTGACACTGACCAAGT -3'-BITEG
STAiR6.6	5'- GGGAATTACTCTGTGCGATT -3'-BITEG
STAiR6.7	5'- TAACGTTACGGCCACGTTAA -3'-BITEG
STAiR6.8	5'- GCGAAACTTTGAGGAAGCTC -3'-BITEG
STAiR6.9	5'- AAACTTCTGTCCAAGGGAGT -3'-BITEG
STAiR6.10	5'- TCTTCTGGTGTGGGAGAATG -3'-BITEG
STAiR6.11	5'- TGTACCCGAGAGAAACTCAC -3'-BITEG
STAiR6.12	5'- CATTTGTAACCGAGCAGACG -3'-BITEG
STAiR15.1	5'- CTAGTGCAGAGGGCTCTTTG -3'-BITEG
STAiR15.2	5'- AACCACATGGGTGGACAGAT -3'-BITEG
STAiR15.3	5'- CTGGCTTGATCCACAGTGAC -3'-BITEG
STAiR15.4	5'- ATGCACCAGAGGAAATTCGG -3'-BITEG
STAiR15.5	5'- CTGACATGAAGATCTGAGCA -3'-BITEG
STAiR15.6	5'- GAATCAAGTTCACATGGGGC -3'-BITEG
STAiR15.7	5'- CTGGAATTGCAGATTGTGGG -3'-BITEG
STAiR15.8	5'- CATCTCCCACAGTTCTAAAC -3'-BITEG
STAiR15.9	5'- CATCTGCTTGCCAAAAGGAG -3'-BITEG
STAiR15.10	5'- AGGAGTGAAGATATGCCCAA -3'-BITEG
STAiR15.11	5'- AAATCCACTAACTCCCTTTC -3'-BITEG
STAiR15.12	5'- AATTTCTACCCACACATTCG -3'-BITEG
STAiR18.1	5'-TTCTCATTGGAACGAGATGACTCAT-3'-BITEG
STAiR18.2	5'-TCCATTCCCAATGATGTACACACGA-3'-BITEG
STAiR18.3	5'-TCAGGCACCGCTTGTCTGGAATGTC-3'-BITEG
STAiR18.4	5'-GAACTGTGCTGTGAAGATCTGAAGA-3'-BITEG
STAiR18.5	5'-CCTGTTTCATCTCCCAGTTATTCAG-3'-BITEG
STAiR18.6	5'-ATATTCGATCAAGTGTGTCATAGAG-3'-BITEG
STAiR18.7	5'-TCAGCTGCATTCCGGCTGTGATCGG-3'-BITEG
STAiR18.8	5'-GGTTGGAACCAGGCCCCAGGGAATC-3'-BITEG
STAiR18.9	5'-CAAATGCAGAGGCCTCAGAGTCCAC-3'-BITEG
STAiR18.10	5'-CATGACCAAAATATCACAGGCAGAC-3'-BITEG
STAiR18.11	5'-CAGACAAATGGGAAACCGACCAGAC-3'-BITEG
STAiR18.12	5'- AATGAAGGACAAGGGATTAAGACAC-3'-BITEG

Supplemental Table 3: Overview of used patient/donor material

Supplemental Table 3: Collection of RNA samples used to evaluate STAiR expression in tumor entities. The table contains clinical information about the used tumorous and healthy tissue samples as it was stated by the manufacturers.

Tissue	Patient matched	Gender	Age	Clinical diagnosis	Number donors	Order number	Manufact urer
Breast	Yes	Female	53	Invasive Ductal Carcinoma, Poorly Differentiated, Metastasize to Armpit Lymph Node	1	R8235086-PP-10	Biochain
Colon	Yes	Male	68	Adenocarcinoma, Moderately Differentiated, Colon Tumor Metastasize to Lymph Node	1	R8235090-PM-10	Biochain
Kidney	Yes	-	-	-	-	R8235142-PM-10	Biochain
Liver	Yes	Male	59	Liver cancer, Liver cancer Metastasize to Lymph Node	1	R8235149-PM-10	Biochain
Lung	Yes	-	-	-	1	R8235152-PM-10	Biochain
Bladder	No	Male	44	Normal	1	R1234010-50	Amsbio
Bladder	No	-	-	Tumor	1	R1235010-10	Amsbio
Prostate	No	Male	24	Normal	1	R1234201-50	Amsbio
Prostate	No	Male	48	Adenocarcinoma of prostate	1	CR560070	Origene

*for kidney, lung and bladder no further information was given by the manufacturer

Supplemental Figure 1: Chromatin-association of STAiRs

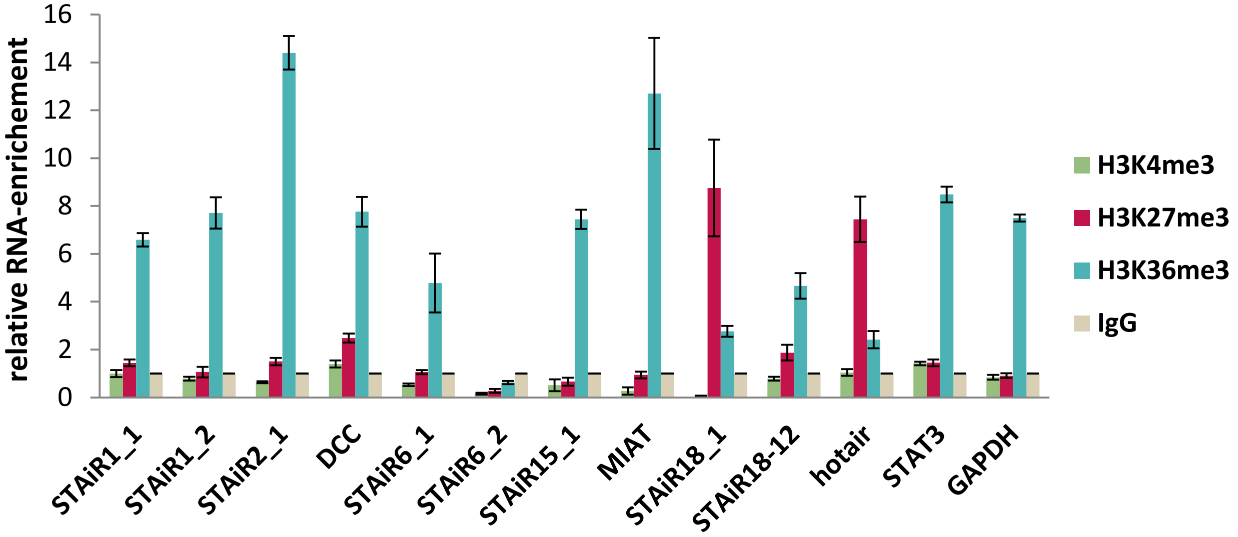

Supplemental Figure 1: Chromatin association of STAiRs. RNA immunoprecipitation was performed with permanently IL-6-treated INA-6 cells using antibodies targeting H3K36me3, H3K4me3, and H3K27me3 as well as IgG as a negative control. RNA was prepared, DNase-digested, and reverse transcribed. RNA enrichment was analyzed by qPCR using gene-specific primers. Samples were normalized to the IgG control.

Supplemental Figure 2: Raw data image files of agarose gels depicted in Figure 3

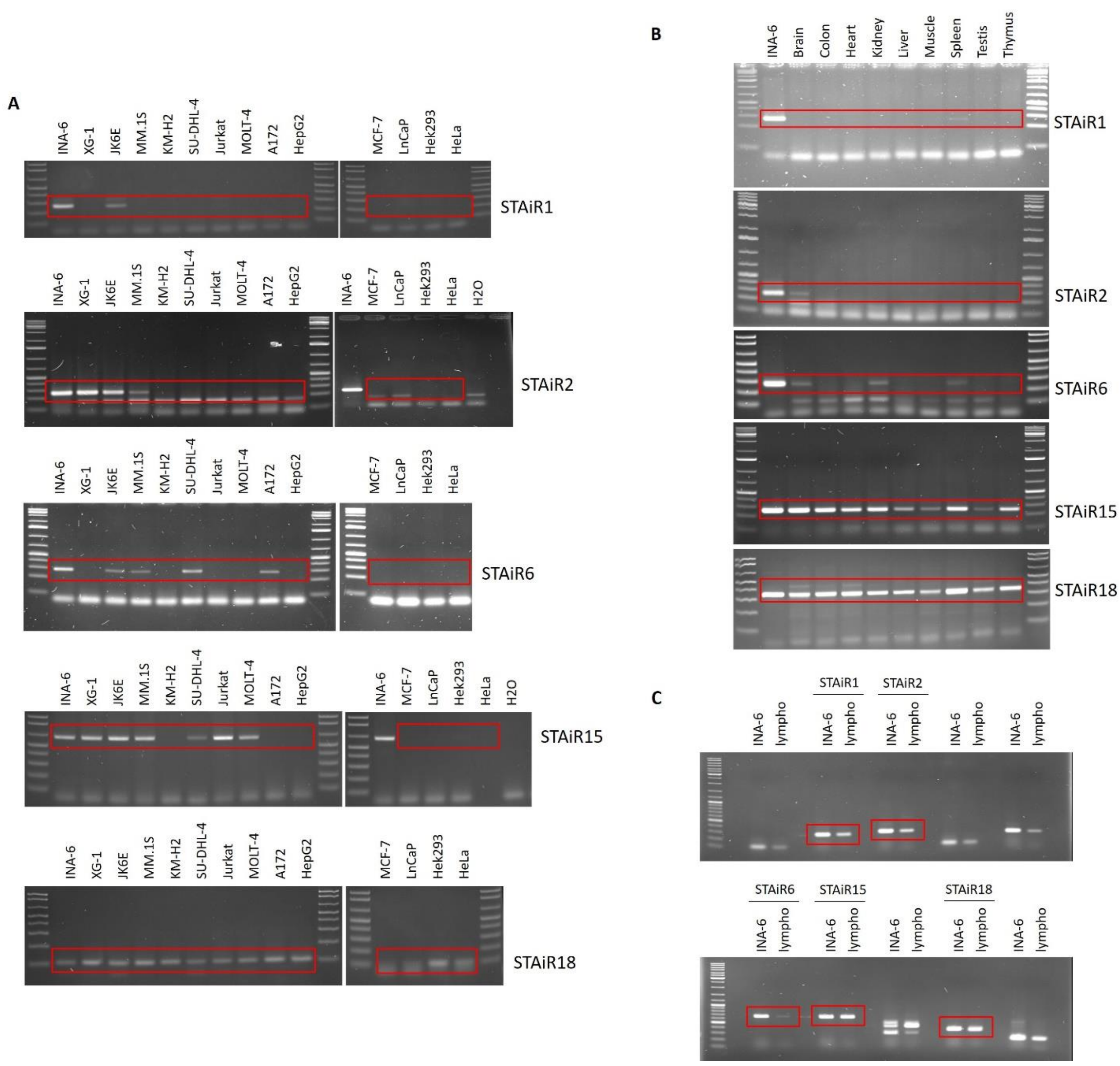

Supplemental Figure 2: Raw data image files depicted in Figure 3 displaying the expression of STAiRs in (A) cell lines and (B) tissues. RNA was isolated from various (A) cell lines and primary lymphocytes (C). DNase-digested RNAs as well as Ambion's FirstChoice® Human Tissue Total RNAs (B) were reverse-transcribed and subjected to standard PCR using primer pairs for primary STAiRs, resulting in product sizes of 264bp for STAiR1, 253bp for STAiR2, 271bp for STAiR6, 252bp and 625bp for STAiR15.1 and STAiR15.2, respectively, as well as 330bp and 106bp for STAiR18.1 and STAiR18.2, respectively. No adjustments of brightness and contrast were made for this figure. Cropped bands marked by a red box were inverted and displayed in Figure 3 of this manuscript.

Supplemental Figure 3: Analysis of STAiR binding motifs by MEME

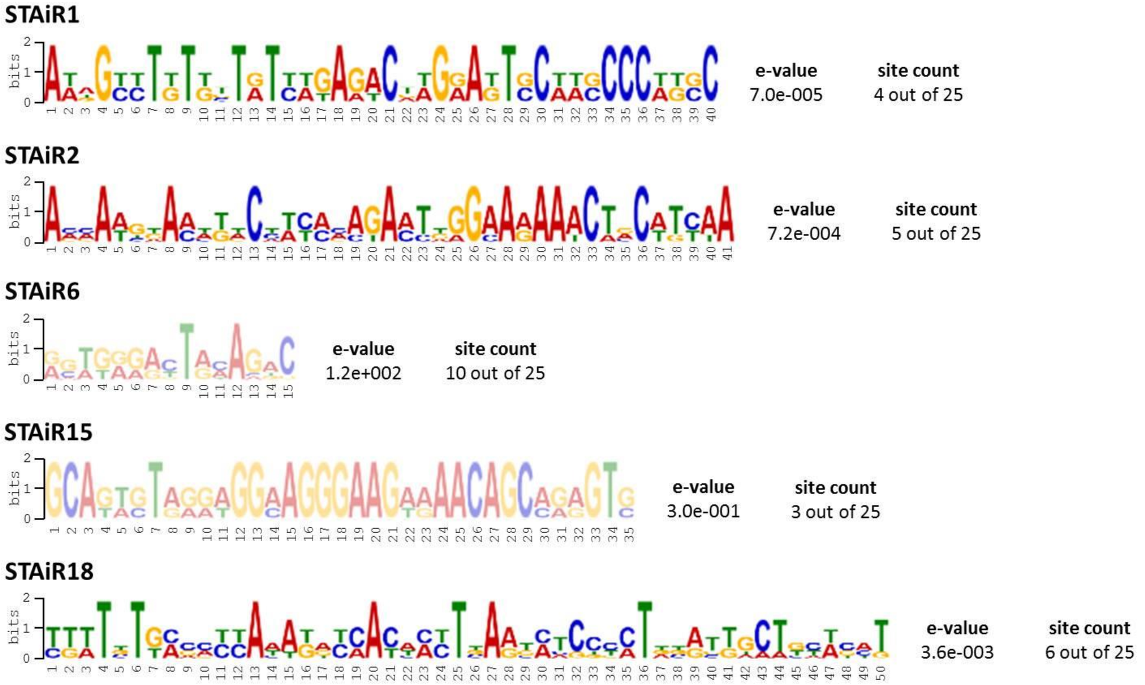

Supplemental Figure 3: Analysis of potential STAiR binding motifs by MEME. Following STAiR-ChIRP-seq, read sequences of the top 25 RNAs specifically interacting with each STAiR compared to the lacZ negative control were analyzed by MEME (Multiple Em for Motif Elicitation) online tool. Compared read sequence lengths varied from 50-200nt. Computed motifs are shown on the left. On the right, e-values for each motif and the number of RNA sequences (out of 25) found to harbor this motif are depicted. Nonsignificant motifs are exposed in transparent.

Supplemental Figure 4: Detection of human DCC protein product in dependence of IL-6 treatment

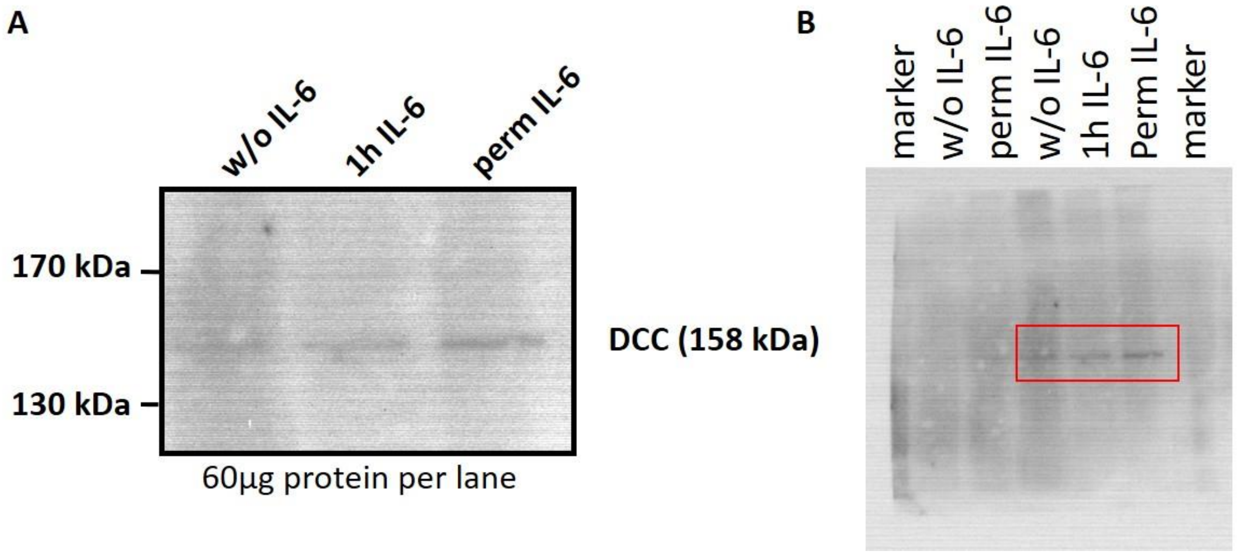

Supplemental Figure 4: Detection of human DCC protein product in dependence of IL-6 treatment (A) and the according raw data image (B). INA-6 cells were either permanently grown in the presence of IL-6 (perm IL-6) or withdrawn from IL-6 for 12 h (w/o IL-6), and subsequently restimulated with IL-6 for 1 h (1h IL-6). Proteins were isolated using RIPA buffer and 60µg protein lysate were separated by SDS-PAGE (8% PAA gel). Proteins were blotted onto a PVDF membrane and visualized using a primary antibody targeting human DCC (abcam ab118918, rabbit) and a secondary swine-anti-rabbit-HRP antibody (DAKO P0217). Detection occurred upon SuperSignal® maximum sensitivity substrate (Thermo #34095) addition with a CCD camera.
